# Overexpression of the β2AR gene improves function and re-endothelialization capacity of EPCs after arterial injury in nude mice

**DOI:** 10.1186/s13287-016-0335-y

**Published:** 2016-05-18

**Authors:** Xiao Ke, Xiao-Rong Shu, Fang Wu, Qing-Song Hu, Bing-Qing Deng, Jing-Feng Wang, Ru-Qiong Nie

**Affiliations:** Department of Cardiology, Sun Yat-sen Memorial Hospital of Sun Yat-sen University, No. 107, Yanjiangxi Road, Guangzhou, China; Guangdong Province Key Laboratory of Arrhythmia and Electrophysiology, Guangzhou, 510120 China; Department of Geriatric, The First Affiliated Hospital of Sun Yat-sen University, Guangzhou, 510080 China

**Keywords:** β2AR, Re-endothelialization, Proliferation, Migration, Endothelial progenitor cells, β2AR/Akt/eNOS

## Abstract

**Background:**

Proliferation and migration of endothelial progenitor cells (EPCs) play important roles in restoring vascular injuries. β2 adrenergic receptors (β2ARs) are widely expressed in many tissues and have a beneficial impact on EPCs regulating neoangiogenesis. The aim of the present study was to determine the effect of overexpressing β2ARs in infused peripheral blood (PB)-derived EPCs on the re-endothelialization in injured vessels.

**Methods:**

Induction of endothelial injury was performed in male nude mice that were subjected to wire-mediated injury to the carotid artery. Human PB-derived EPCs were transfected with an adenovirus serotype 5 vector expressing β2AR (Ad5/β2AR-EPCs) and were examined 48 h later. β2AR gene expression in EPCs was detected by real-time polymerase chain reaction and Western blot analysis. In vitro, the proliferation, migration, adhesion, and nitric oxide production of Ad5/β2AR-EPCs were measured. Meanwhile, phosphorylated Akt and endothelial nitric oxide synthase (eNOS), which are downstream of β2AR signaling, were also elevated. In an in vivo study, CM-DiI-labeled EPCs were injected intravenously into mice subjected to carotid injury. After 3 days, cells recruited to the injury sites were detected by fluorescent microscopy, and the re-endothelialization was assessed by Evans blue dye.

**Results:**

In vitro, β2AR overexpression augmented EPC proliferation, migration, and nitric oxide production and enhanced EPC adhesion to endothelial cell monolayers. In vivo, when cell tracking was used, the number of recruited CM-DiI-labeled EPCs was significantly higher in the injured zone in mice transfused with Ad5/β2AR-EPCs compared with non-transfected EPCs. The degree of re-endothelialization was also higher in the mice transfused with Ad5/β2AR-EPCs compared with non-transfected EPCs. We also found that the phosphorylation of Akt and eNOS was increased in Ad5/β2AR-EPCs. Preincubation with β2AR inhibitor (ICI118,551), Akt inhibitor (ly294002), or eNOS inhibitor (L-NAME) significantly attenuated the enhanced in vitro function and in vivo re-endothelialization capacity of EPCs induced by β2AR overexpression.

**Conclusions:**

The present study demonstrates that β2AR overexpression enhances EPC functions in vitro and enhances the vascular repair abilities of EPCs in vivo via the β2AR/Akt/eNOS pathway. Upregulation of β2AR gene expression through gene transfer may be a novel therapeutic target for endothelial repair.

## Background

Coronary atherosclerotic heart diseases are the major cause of mortality worldwide in cardiovascular disease (CVD), yet current therapies only delay disease progression and improve lifestyle without addressing the fundamental problem of tissue loss. Several studies have shown that endothelial dysfunction is an early marker of atherosclerosis, and the activation of inflammatory processes and abnormalities in vascular homeostasis have been suggested to contribute to the development of atherosclerotic vascular disease [1]. Accumulating evidence indicates that the balance between endothelial injury and repair is a key component of atherosclerosis [2], and maintaining endothelial integrity is crucially important to preventing the initiation and development of atherosclerosis, coronary heart disease, and postangioplasty restenosis [3]. Thus, accelerated re-endothelialization might prevent the early stages of atherosclerosis and restenosis after angioplasty.

Endothelial progenitor cells (EPCs) mobilized from bone marrow into the peripheral blood (PB) have been shown to play an important role for vascular regeneration, endothelial repair, and replacement of dysfunctional endothelium by incorporating into the site of vessel injury, differentiating into endothelial cells (ECs), and releasing paracrine factors [4, 5]. Transplantation of EPCs is currently under intensive investigation in animal models and clinical research and have become a major focus of CVD treatment to accelerate re-endothelialization [6]. However, the beneficial effects of this practice have not been observed in patients with coronary artery disease, because the reparative capacity of EPCs appears to be limited by their poor survival environment [7]. Therefore, attempts to improve the function of transplanted EPCs with gene modifications may facilitate the repair of damaged endothelia and accelerate re-endothelialization.

Various molecules may be involved in the processes by which EPCs home to and restore damaged endothelium, such as nitric oxide (NO), stromal cell-derived factor 1a (SDF-1a), and vascular endothelial growth factor (VEGF). It is widely accepted that β-adrenoceptors exist on ECs and contribute to the regulation of vasomotor tone. Moreover, it is the β2 adrenergic receptor (β2AR), the most abundant βAR in the vasculature, that mediates the NO involved in relaxing vascular tone [8]. β2ARs, G protein-coupled receptors, are activated by adrenergic catecholamine to promote a series of intracellular signal transduction pathways that lead to multiple cell-specific responses [9]. It has been demonstrated that β2ARs are strongly expressed on EPCs and also mediate the homing and neovascularization capacity of EPCs to areas of ischemia [9]. Accordingly, the β2AR gene may be a valuable molecular target for gene therapies that use EPCs. However, β2ARs have not previously been shown to accelerate vascular repair via re-endothelialization mediated by EPCs. Thus, we sought to determine whether β2AR gene transfer mediates the functional properties of EPCs during vascular injury.

In this study, we tested the therapeutic potential of β2AR gene transfer in EPCs by infusing transfected cells into nude mice after we induced an endothelial denudation injury. We also investigated the β2AR-mediated Akt/endothelial nitric oxide synthase/NO (Akt/eNOS/NO) signaling pathway that is related to both in vitro and in vivo biology of EPCs. These data demonstrate that the β2AR has an important role in EPC migration at the vascular injury site, and upregulating β2AR expression is a potential new therapeutic strategy that may improve the efficiency of EPC-induced re-endothelialization.

## Methods

### Ethics

The experimental research on humans in this study was performed in compliance with the Helsinki Declaration. All recruited patients consented to participate in this trial and to contribute their trial data for non-commercial purposes. The protocol of this trial was externally reviewed and approved by an anonymous independent ethical review committee to ensure that there were no serious ethical concerns. The animal procedures in this study complied with the Animal Care and Use Ethics Committees of Sun Yat-Sen University.

### EPC culture and characterization

EPCs were isolated and cultured according to previously described methods [10, 11]. Briefly, PB mononuclear cells (MNCs) were isolated from healthy subjects (males from 25 to 35 years of age) by using Histopaque-1077 density gradient centrifugation at 400 *g* for 30 min. The collected MNCs were washed three times with phosphate-buffered saline (PBS) (Jingmei Bio Tech Co. Ltd., Shenzhen, China). After the cells were purified, the MNCs were cultured on fibronectin-coated six-well plates in endothelial basal medium-2 (EBM-2) (CC-4176; Lonza, Basel, Switzerland) supplemented with EGM-2 Bullte Kit (Lonza) and 20 % fetal bovine serum (FBS) (Gibco, now part of Thermo Fisher Scientific, Waltham, MA, USA). After 4 days in culture, the non-adherent cells were abandoned. Adherent cells were cultured for 7 days and then were used for the following experiments.

EPCs were defined as cells that were dually positive when stained by using 1,1′-dioctadecyl-3,3,3′,3′-tetramethylindocarbocyanine (DiI)-acetylated low-density lipoprotein (ac-LDL) (20 μg/ml; Invitrogen, Carlsbad, CA, USA) and fluorescein isothiocyanate (FITC)-labeled BS-1 lectin (10 μg/ml; Sigma-Aldrich, St. Louis, MO, USA). Cultured EPCs were incubated with DiI-ac-LDL for 3 h at 37 °C; the cells then were washed in PBS, fixed in 4 % (vol/vol) paraformaldehyde (PFA) for 30 min, and incubated with FITC-labeled BS-1 lectin for 1 h. The cells were washed again and then incubated with 4′,6-diamidino-2-phenylindole (DAPI), a nuclear counterstain. Double-positive cells were observed with a fluorescence microscope (×200 magnification; Olympus, Tokyo, Japan). Cells demonstrating double-positive fluorescence were identified as differentiating EPCs.

### Flow cytometric analysis

The expression of endothelial marker proteins was examined in the cultured EPCs by using flow cytometric analysis with phycoerythrin (PE)-labeled monoclonal mouse anti-human antibodies recognizing CD31 (BD Pharmingen, San Diego, CA, USA), von Willebrand factor (vWF) (BD Pharmingen), kinase-insert domain receptor (KDR) (R&D Systems, Minneapolis, MN, USA), and CD14 (BD Pharmingen). To identify the cells that expressed these surface antigens, the EPCs were incubated for 40 min at 4 °C in a volume of 100 μl of solution containing an appropriate amount of PE-labeled antibody or corresponding IgG isotype control. At least 1 × 10^5^ EPCs were acquired by using a flow cytometer (Beckman-Coulter, Fullerton, CA, USA).

### Immunofluorescence

To characterize the expression of EC markers, EPCs were grown in fibronectin-coated six-well plates, and immunofluorescence analysis was performed by using rabbit polyclonal antibody against β2AR (Abcam, Cambridge, MA, USA) and mouse monoclonal antibody (mAb) against eNOS (Cell Signaling Technology, Boston, MA, USA). Briefly, the cells were washed in cold PBS three times and fixed in 4 % PFA for 30 min. Then the cells were washed again with PBS three times for 5 min each and incubated in 3 % bovine serum albumin (BSA) in PBS for 1 h. The cells were incubated with primary antibodies (anti-eNOS, anti-β2AR diluted 1:100 with 3 % BSA in PBS) at room temperature for 1 h. After the cells were washed three times for 5 min each in PBS on a shaker, the cells were exposed to goat anti-rabbit IgG (H + L) (catalog no. A-11011; Life Technologies, Carlsbad, CA, USA) and goat anti-mouse IgG (H + L) (catalog no. A-11011; Life Technologies) secondary antibodies for 1 h in the dark. The cells were washed again and incubated with DAPI to stain the EPC nuclei. Images were acquired by using a fluorescence microscope (×200 magnification; Olympus).

### EPC gene transfer

An adenovirus sero-type 5 (Ad5) vector expressing the human β2AR gene (Ad5/β2AR) or enhanced green fluorescent protein (Ad5/EGFP) was used for gene delivery (purchased from GeneChem Company Ltd., Shanghai, China). To establish the appropriate virus concentration for adenoviral gene transfer into EPCs, the effectiveness of different multiplicities of infection (MOIs) was evaluated in accordance with the instructions of the adenovirus manufacturer. Briefly, after the EPCs were cultured for 7 days, they were transduced with Ad5/β2AR and Ad5/EGFP in serum-free culture medium (MOI of approximately 500). The viruses were removed, and the cells were washed with PBS and incubated with EPC medium for another 48 h before subsequent experiments.

### Real-time polymerase chain reaction and Western blot analysis

Total cellular RNA was isolated by using TRIzolreagent (Invitrogen). Double-stranded cDNA was synthesized by using an M-MLV Reverse Transcriptase cDNA Synthesis Kit (TaKaRa, Kusatsu, Shiga, Japan). Quantitative polymerase chain reaction (PCR) was carried out with Light Cycler 480 SYBR Green I Master Mix (Roche Diagnostics, Risch-Rotkreuz, Switzerland) in a Light Cycler 480 System. The cycling protocol for the PCR was as follows: 95 °C for 5 min, followed by 45 cycles of 95 °C for 10 s, 60 °C for 10 s, and 72 °C for 20 s. The primers used were as follows: β2AR: 5′-ATGGTGTGGATTGTGTCAGG-3′ (forward) and 5′-CAGGTCTCATTGGCATAGCA-3′ (reverse) and glyceraldehyde 3-phosphate dehydrogenase (GAPDH): 5′-GGTGGTCTCCTCTGACTTCAACA-3′ (forward) and 5′-GTTGCTGTAGCCAAATTCGTTGT-3′ (reverse).

EPCs were lysed with cell lysis buffer (Cell Signaling Technology) in accordance with the instructions of the manufacturer. Cell lysates were quantified by bicinchoninic acid (BCA) methods in accordance with the instructions of the manufacturer (Sangon Biotechnology, Shanghai, China). In total, 50 μg protein was subjected to SDS-PAGE and then transferred to polyvinylidene fluoride membranes. The following antibodies were used: rabbit anti-β2AR antibody (1:1000; Abcam, Cambridge, MA, USA), Phospho-Akt (Ser473) rabbit mAb (1:1000; Cell Signaling Technology), Phospho-eNOS (Ser1177) rabbit mAb (1:1000; Cell Signaling Technology), eNOS (49G3) rabbit mAb (1:1000; Cell Signaling Technology), Akt (C67E7) rabbit mAb (1:1000; Cell Signaling Technology), SDF-1 antibody (1:1000; Cell Signaling Technology), CXCR4(H-118) (1:1000; Santa Cruz Biotechnology, Inc., Dallas, TX, USA), and GAPDH (14C10) rabbit mAb (1:1000; Cell Signaling Technology). Proteins were visualized with horseradish peroxidase-conjugated anti-rabbit IgG (1:5000; Cell Signaling Technology). To detect the effect of stimulation of PB-derived EPCs with the selective β2AR agonist fenoterol (FENO) on the phosphorylation of Akt and eNOS, EPCs were pre-incubated with 10^−8^ M FENO (Sigma-Aldrich) for 6 h before proteins were harvested.

### EPC proliferation and NO production

The effect of β2AR gene transfer into EPCs on cell proliferation was assessed by CCK8 (Dojindo Molecular Technologies, Kumamoto, Japan). EPCs were transduced with Ad5/β2AR or Ad5/EGFP, or they were not transduced (control). The EPCs were reseeded on 96-well plates. Briefly, the EPCs were seeded in 96-well plates (5 × 10^3^ cells per well) in EBM-2 (Lonza, CC-4176) supplemented with 1 % FBS for 24 h. The medium then was replaced with 100 μl of fresh medium containing 10 μl of CCK8 solution, and the cells were incubated for another 2 h. The absorbance of each well then was determined at 450 nm by using an Infinite F200 Multimode plate reader. We used 0.3 μM ICI118,551 (Sigma-Aldrich), 10 μM LY294002 (Calbiochem, now part of EMD Millipore, Billerica, MA, USA), and 100 μM L-NAME (Calbiochem) to inhibit β2AR, Akt, and eNOS, respectively. The EPCs were preincubated with inhibitor for 30 min before FENO (10^−8^ M) stimulations.

NO secretion by EPCs was measured as the generation of nitrite. The cells were cultured with EBM-2 (growth factor-free) for 48 h after gene transfer. The supernatants were assayed to determine the level of NO by using a NO assay kit by the nitrate reductase method (Nanjing Jiancheng Institute of Biological Engineering, Nanjing, China).

### In vitro EPC migration assays

EPC migration assays were performed by using a Transwell system (Corning Costar, Tewksbury, MA, USA) with 8-μm polycarbonate filter inserts in 24-well plates. Briefly, a total of 2 × 10^4^ EPCs were suspended in 250 μl of EBM-2 medium supplemented with 1 % FBS, and the cells were incubated in the upper chamber for 30 min for each group. These groups included the control group, Ad5/β2AR-EPC group, and Ad5/EGFP-EPC group, which were not pretreated, and the Ad5/β2AR-EPC group and Ad5/EGFP-EPC group, which were pretreated with ICI118,551, LY294002, and L-NAME. The lower compartment of the modified Boyden chamber was placed in a 24-well culture plate in which each well was filled with 500 μl of EBM-2 supplemented with PBS or FENO (10^−8^ M). After the cells were cultured for 6 h, the cells that had migrated into the lower chamber were stained with DAPI. The transmigrated cells were randomly counted by an independent investigator who was blinded to treatments.

### In vitro EPC adhesion assays

A monolayer of human umbilical vein endothelial cells (HUVECs) was prepared 48 h before the assay by plating 2 × 10^5^ cells in each well of a four-well plate. The HUVECs were pretreated with or without 1 ng/ml tumor necrosis factor-α (TNF-α) (PeproTech, Rocky Hill, NJ, USA) for 12 h. Then 1 × 10^5^ CM-DiI (CellTracker™ CM-DiI, Invitrogen)-labeled EPCs was added to each well, and the cells were incubated for 3 h at 37 °C. The non-attached cells were gently removed with PBS, and the adherent EPCs were fixed in 4 % PFA and randomly counted by an independent investigator who was blinded to treatments.

### Animal model and in vivo re-endothelialization assay

The carotid artery injuries and EPC transplantation were performed by using previously described methods [12, 13]. Male NRMInu/nu athymic nude mice (The Laboratory Animal Center of Sun Yat-sen University, Guangzhou, China) that were 6 to 8 weeks old were injected with human PB-derived EPCs. The animals were anaesthetized with ketamine (100 mg/kg intraperitoneally) and xylazine (5 mg/kg intraperitoneally). The surgeries were performed by using a stereoscopic microscope. The left carotid artery was exposed via a midline incision on the ventral side of the neck. The bifurcation of the carotid artery was located, and two ligatures were placed around the external carotid artery, which then was tied off with the distal ligature. An incision hole was made between the ligatures to introduce the denudation device. A curved flexible wire (0.35-mm diameter) was introduced into the common carotid artery and passed over the lining of artery three times to denude the endothelium. The wire then was removed, and the external carotid artery was tied off proximal to the incision hole with the proximal ligature.

EPCs (1 × 10^6^ cells) that had been cultured for 7 days were resuspended in 100 μl of pre-warmed PBS (37 °C) and were transplanted 3 h after carotid artery injury via tail vein injection with a 27-G needle. The same volume of PBS was injected into placebo mice as a control. Three days after carotid artery injury, endothelial regeneration was evaluated by staining denuded areas with 50 μl of 5 % Evans blue dye via tail vein injection. To examine the homing of transplanted EPCs to the site of the injured carotid vessel, labeled EPCs (1 × 10^6^) were incubated with CM-DiI (Cell Tracker™ CM-DiI; Invitrogen) in accordance with the instructions of the manufacturer. CM-DiI-labeled EPCs incorporated in the injured vessels were quantitatively analyzed under a fluorescence microscope (Olympus BX51).

### Statistical analysis

All results are expressed as the mean ± standard error of the mean. Statistical significance was evaluated by means of Student’s *t* test or analysis of variance. A *P* value of less than 0.01 was considered to denote statistical significance. All statistical analyses used SPSS statistical software (SPSS version 13.0; IBM Corporation, Armonk, NY, USA).

## Results

### Characterization of EPCs and endogenous expression of β2AR on EPCs

**Fig. 1:**
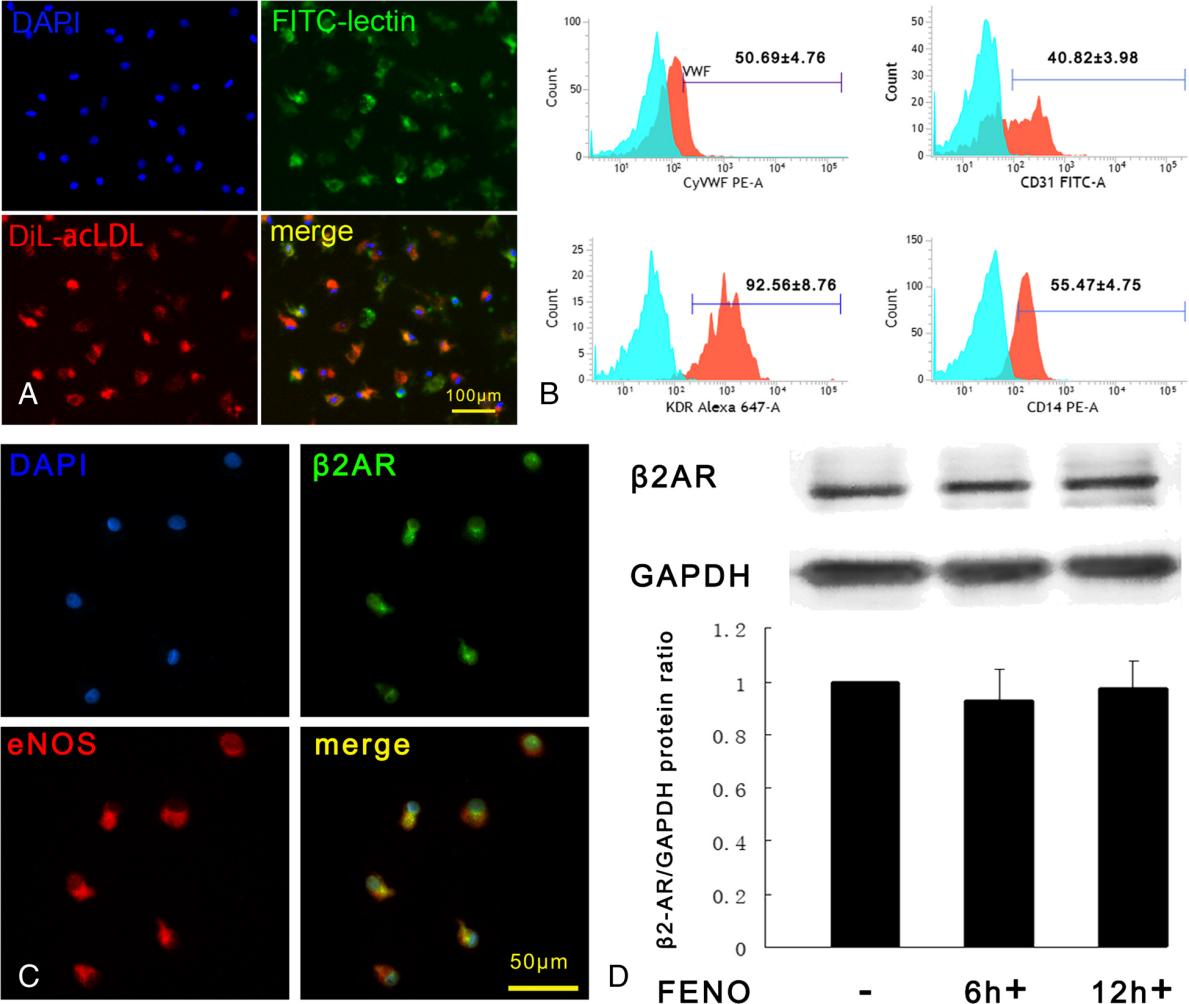
Characterization of EPCs and endogenous expression of β2AR on EPCs. **a** Representative photographs of EPCs at 7 days that were labeled with DAPI (*blue*), FITC-labeled BS-1 lectin (*green*), and DiI-acLDL uptake (*red*). Double-labeled cells were identified as EPCs (*yellow*) (×200). **b** Cells were tested for the ability to express the endothelial markers CD31, vWF, KDR, and CD14 by using flow cytometry analysis (IgG isotype control is shown in *blue*, *n* = 4 per group). **c** The expression of β2AR protein was confirmed on EPC surfaces by using immunofluorescence. The expression of β2AR (*green*) and eNOS (*red*) and colocalization of both receptors on EPCs (*yellow*) are shown. **d** Representative photographs and quantitative analyses of the β2AR protein expressed on EPCs after stimulation with FENO. The results show that β2AR expression was not changed in the EPCs that were stimulated by using FENO. *DAPI* 4‘,6-diamidino-2-phenylindole, *DiI-ac-LDL* DiI-labeled acetylated low-density lipoprotein, *eNOS* endothelial nitric oxide synthase, *EPC* endothelial progenitor cell, *FENO* fenoterol, *FITC* fluorescein isothiocyanate, *KDR* kinase-insert domain receptor, *vWF* von Willebrand factor, *β2AR* β2 adrenergic receptor

Recently, EPCs were classified into two distinct types: early and late EPCs. Early EPCs appear after 5 to 7 days, and late EPCs appear after 14 to 21 days [14]. A beneficial effect on vascular repair after injury has been shown for early EPCs [15]. After 7 days of culture on fibronectin-coated plates, PB-derived EPCs had a spindle-shaped morphology. Cellar immunostaining showed that most of the adherent cells have double-positive staining for the uptake of DiI-ac-LDL and for the binding of FITC-lectin, indicating that these cells possess the functional properties of ECs (Fig. 1a). In addition, flow cytometric analysis of EC antigens revealed that 40.82 ± 3.98 % of the adherent cells were positive for CD31, 50.69 ± 4.76 % for vWF, 96.56 ± 8.76 % for VEGF (VEGFR2/KDR), and 55.47 ± 4.75 % for the monocytic maker CD14 (Fig. 1b). All of these characteristics indicate that the cultured adherent cells were appropriately identified as EPCs, as previously described [12, 16]. We performed immunocytochemistry for colocalization of β2ARs and the EPC marker eNOS. Fluorescence microscopy revealed that β2ARs were found to localize to the cell membrane of EPCs, and these results were confirmed by using a positive marker, eNOS, which is also expressed in EPCs (Fig. 1c). In addition, β2AR expression on the EPCs did not increase when the cells were stimulated with the selective β2AR agonist FENO for 6 or 12 h, as shown in the Western blot analyses. These results are consistent with the findings of Galasso et al. (Fig. 1d) [9].

### Overexpression of β2AR in transfected EPCs

**Fig. 2:**
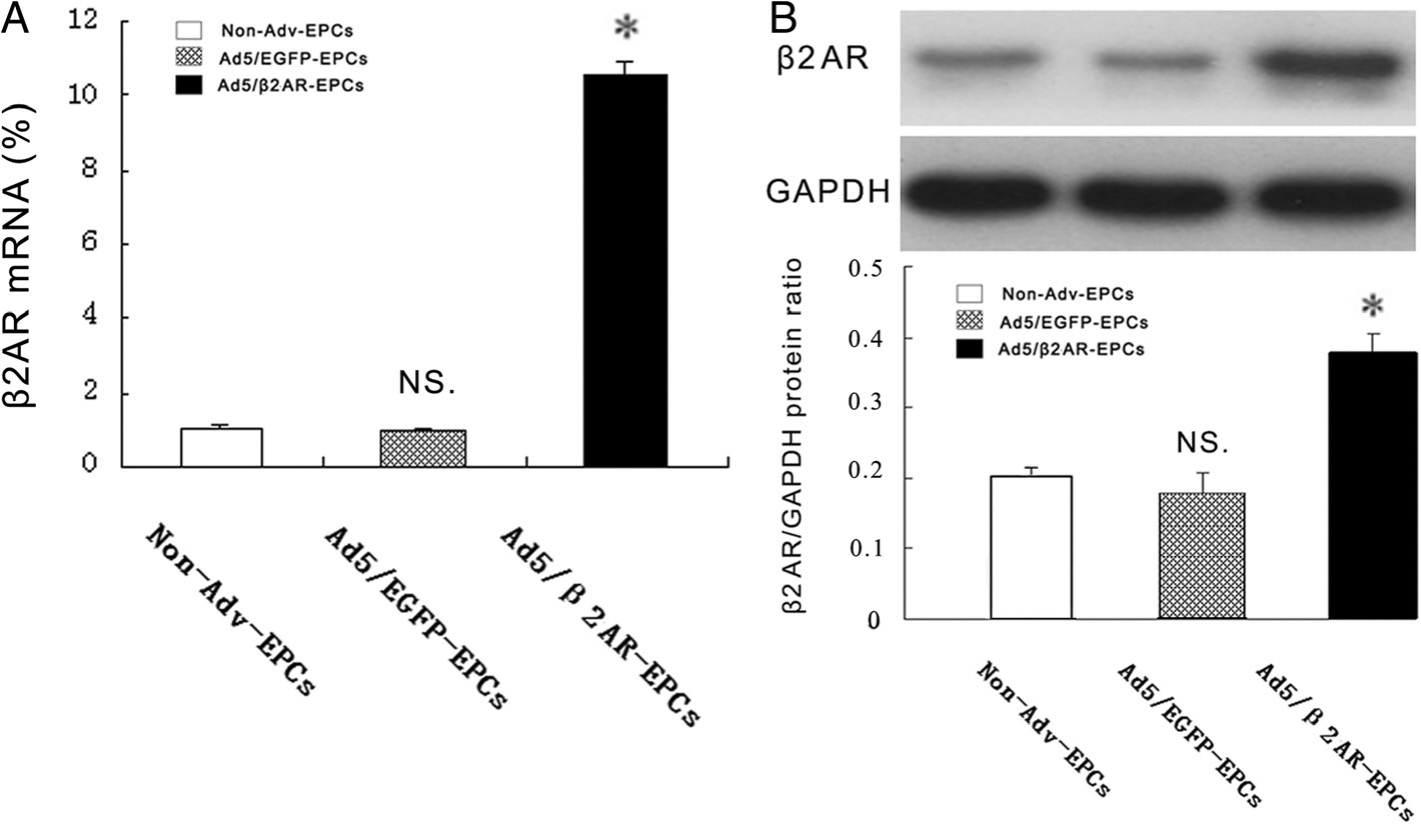
Overexpression of β2AR in transfected EPCs. **a** β2AR mRNA levels were measured in non-Adv EPCs, Ad5/EGFP EPCs, and Ad5/β2AR EPCs by using real-time polymerase chain reaction and normalized against β-actin (*n* = 5 per group). *P < 0.01 versus non-Adv EPCs or Ad5/EGFP EPCs. *NS* not significant versus non-Adv-EPCs. **b** β2AR protein levels were significantly higher in the Ad5/β2AR EPC group than in the non-Adv EPC or Ad5/EGFP-EPC group. *P < 0.01 versus non-Adv EPCs or Ad5/EGFP EPCs. *NS* not significant versus non-Adv-EPCs (*n* = 5 per group). *Ad5* adenovirus serotype 5, *EGFP* enhanced green fluorescent protein, *EPC* endothelial progenitor cell, *β2AR* β2 adrenergic receptor

Adenovirus effectively mediated the transfection of the β2AR gene into EPCs. To upregulate the β2AR gene in EPCs, we transduced EPCs by using Ad5/β2AR gene transfection. At 24 h after transfection, EGFP expression was detected by fluorescent microscopy, and the transfection efficiency was approximately 80 % (data not shown). At 48 h after transfection, our results showed that β2AR mRNA was significantly increased in the Ad5/β2AR-EPC group compared with the Ad5/EGFP-EPC group or in controls by using real-time PCR (9.73 ± 3.56 versus 0.89 ± 0.65 or 1.00 ± 0.47; *P* < 0.01). These results were further confirmed by using Western blot analysis (0.40 ± 0.03 versus 0.18 ± 0.03 or 0.2 ± 0.01; *P* < 0.01) (Fig. 2a, b).

### β2AR gene transfer in EPCs upregulates cell proliferation, migration and adhesion; NO production; SDF-1/CXCR4 expression in vitro

**Fig. 3:**
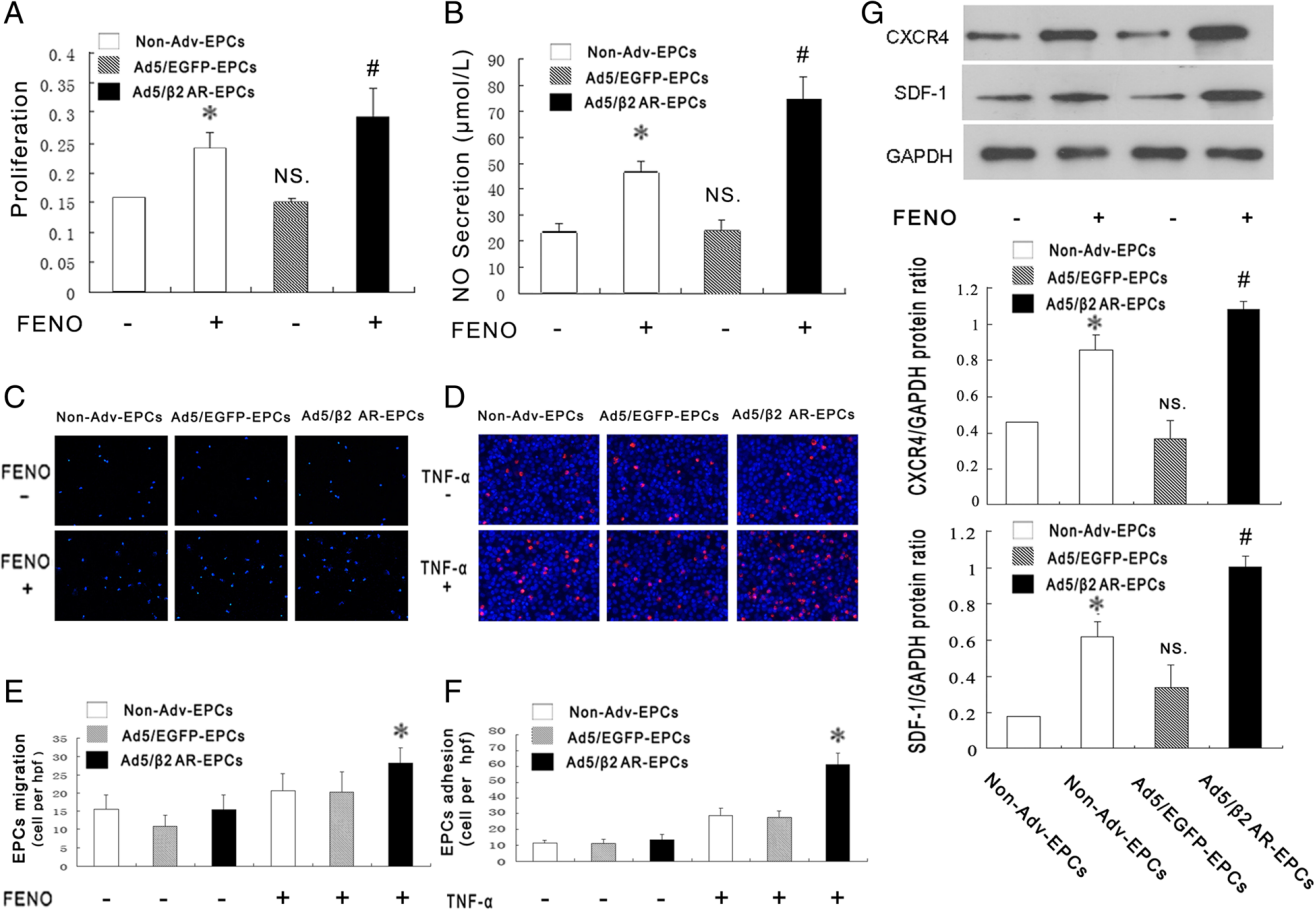
β2AR gene transfer in EPCs upregulates cell proliferation, migration, and adhesion; NO production; and SDF-1/CXCR4 expression in vitro. **a** Proliferation was measured in cultured EPCs by using CCK8 assays after 24 h of stimulation with FENO (10^−8^ M). The rate of proliferation in the Ad5/β2AR EPC group was significantly higher than the rate in the non-Adv-EPC, non-Adv- EPC + FENO, or Ad5/EGFP-EPC groups (0.29 ± 0.05, 0.16 ± 0.00, 0.24 ± 0.03, and 0.15 ± 0.01, respectively; *P < 0.01 versus non-Adv EPCs or Ad5/EGFP-EPCs, ^#^ *P* < 0.01 versus non-Adv-EPCs, non-Adv-EPCs + FENO, or Ad5/EGFP-EPCs; *n* = 5 per group). **b** NO secretion was assayed in EPCs by using the nitrate reductase method after β2AR gene transfer. The production of NO was significantly higher in the Ad5/β2AR-EPCs than in the non-Adv-EPCs, non-AdvEPCs + FENO, or Ad5/EGFP-EPCs (74.42 ± 8.68, 24.01 ± 2.68, 46.77 ± 4.08, and 24.24 ± 3.71, respectively; *P < 0.01 versus non-Adv-EPCs or Ad5/EGFP-EPCs, and ^#^ *P* < 0.01 versus non-Adv-EPCs, non-Adv-EPCs + FENO, or Ad5/EGFP-EPCs; *n* = 5 per group). **c** and **e** Quantitative analyses (**c**) and representative photographs (**e**) showing the migratory activity observed in EPCs (*P < 0.01 versus non-Adv-EPCs + FENO or Ad5/EGFP EPCs + FENO; *n* = 5 per group). **d** and **f** Quantitative analyses (**d**) and representative photographs (**f**) of CM-DiI-labeled EPCs that adhered to human umbilical vein endothelial cells with or without stimulation with *TNF-α* (1 μg/ml; *P < 0.01 versus non-Adv-EPCs + TNF-α or Ad5/EGFP-EPCs + TNF-α; *n* = 5 per group). **g** Overexpression of β2AR increased the expression of SDF-1 and CXCR4 in EPCs. Representative photograph and quantification analysis for SDF-1 and CXCR4 expression in EPCs (*P < 0.01 versus non-Adv EPCs without FENO activation, ^#^ *P* < 0.01 versus non-Adv-EPCs with FENO activation, and NS versus non-Adv-EPCs without FENO activation, *n* = 5 per group). *Ad5* adenovirus serotype 5, *DiI-ac-LDL* DiI-labeled acetylated low-density lipoprotein, *EGFP* enhanced green fluorescent protein, *EPC* endothelial progenitor cell, *FENO* fenoterol, *NO* nitric oxide, *NS* not significant, *SDF-1a* stromal cell-derived factor 1a, *TNF-α* tumor necrosis factor-α, *β2AR* β2 adrenergic receptor

We used CCK8 assays to examine whether overexpressing β2AR affected proliferation in EPCs that were stimulated with FENO. The rate of proliferation was significantly higher in the Ad5/β2AR-transduced EPCs than in the Ad5/EGFP-transduced EPCs or the non-transduced EPCs (Fig. 3a). We also detected NO production by analyzing NO levels in the conditioned media at 48 h after β2AR gene transfer. As shown in Fig. 3b, the level of NO production was higher in the Ad5/β2AR-transduced EPCs than in the Ad5/EGFP-transduced EPCs or the non-transduced EPCs.

In the migration assays, there were no differences in basal migration capacity among Ad5/β2AR-transduced EPCs, Ad5/EGFP-transduced EPCs, and non-transduced EPCs. However, the rate of EPC migration was significantly higher in Ad/β2AR-transduced EPCs that were induced by FENO stimulation than in the Ad5/EGFP transduced EPCs and non-transduced EPCs that were stimulated by FENO (28.2 ± 4.2 versus 20.2 ± 5.5 or 20.6 ± 4.6; *P* < 0.01) (Fig. 3c-e).

TNF-α can enhance the expression of adhesion molecules in ECs [16]. To determine the function of β2AR in EPC adhesion, we investigated its role in the adhesion of EPCs cultured on mature HUVEC monolayers. Similar to the results of the migration assay, without TNF-α stimulation, there was no difference in the adhesiveness of EPCs cultured on HUVECs among the Ad5/β2AR-transduced, Ad5/EGFP-transduced, and non-transduced EPC groups. However, when the HUVECs were activated with TNF-α, the adhesive ability of the Ad5/β2AR-transduced EPCs was greater than the adhesive ability of the Ad5/EGFP-transduced EPCs and the non-transduced EPCs (61.2 ± 7.2 versus 27.4 ± 4.1 or 28.6 ± 4.9; *P* < 0.01) (Fig. 3d-f). Therefore, these data suggested that β2AR mediated the biological function of EPCs in vitro and that β2AR overexpression would increase the regulatory capacity of EPCs.

The chemokine stromal-derived factor (SDF-1) and its unique receptor CXCR4 (SDF-1 and CXCR4 axis) play an important role invasculogenesis, neovascularization, and re-endothelialization [17, 18]. SDF-1 and CXCR4 are also involved in the biological functions of EPCs, including migration, adhesion, mobilization, and homing. Interestingly, in our study, Western blot analysis revealed that SDF-1 and CXCR4 levels were significantly higher at both time points in the cells that were stimulated with FENO than in the control EPCs that were not stimulated with FENO, and this difference was stronger in the Ad5/β2AR-transduced EPC group (Fig. 3g). These results suggest that β2AR stimulation promotes the expression of members of the SDF-1/CXCR4 axis in EPCs.

### β2AR gene transfer increases the re-endothelialization capacity of EPCs in vivo

**Fig. 4:**
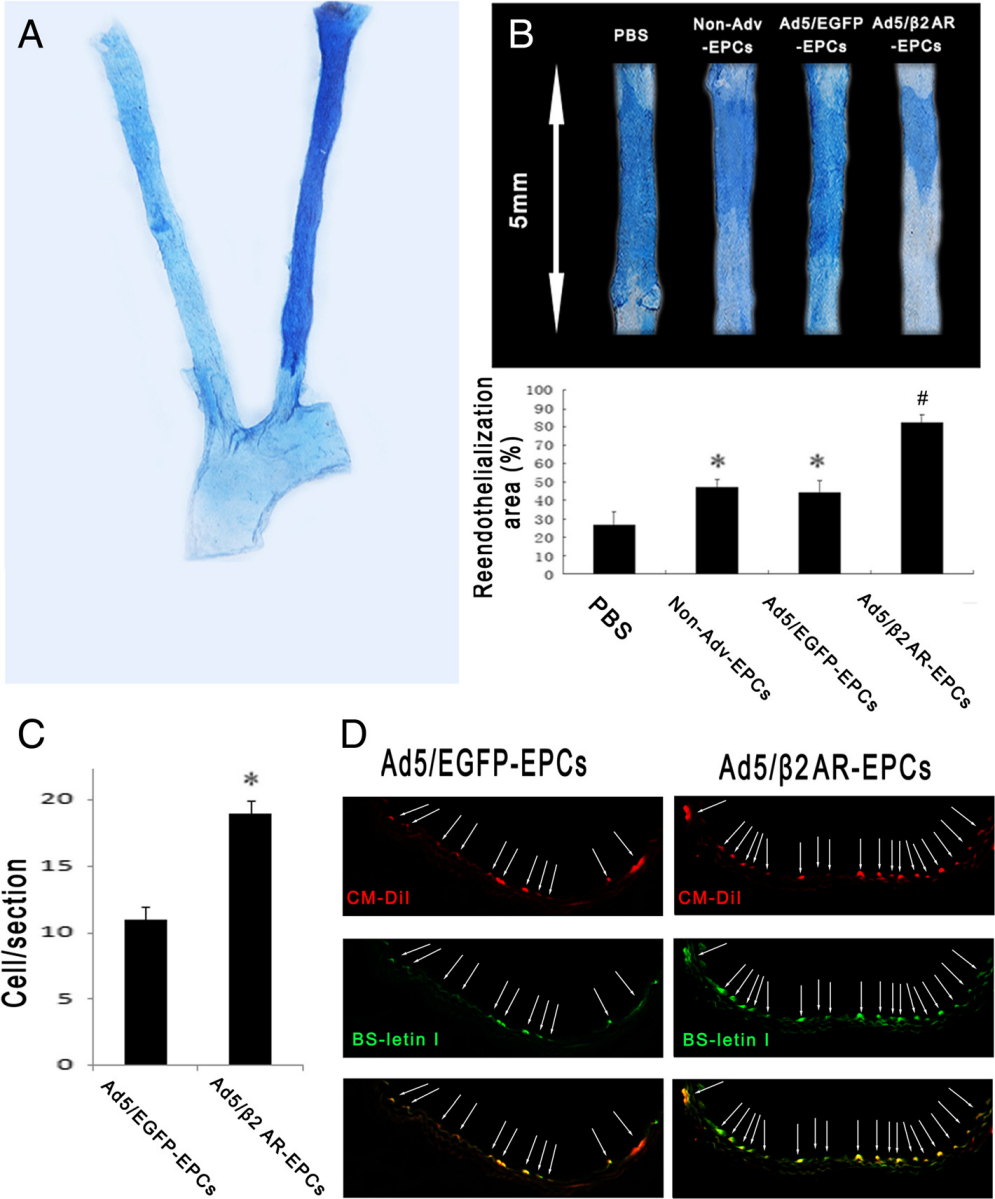
β2AR gene transfer increases the re-endothelialization capacity of EPCs in vivo. Ad5/β2AR gene transfer contributed to the re-endothelialization of injured carotid arteries. **a** Evans blue staining was used to identify segments of denuded endothelium immediately after nude mice were subjected to wire-mediated carotid artery injury. Representative photograph shows an injured artery and a contralateral uninjured artery. **b** The transplantation of EPCs that underwent Ad5/β2AR gene transfer resulted in a higher area of re-endothelialization than was observed in the PBS-injected and non-Adv EPC transplantation group (*n* = 5) or the Ad5/EGFP EPC group (*n* = 5) (*P < 0.01 versus the PBS group, ^#^ *P* < 0.01 versus non-Adv-EPCs, or Ad5/EGFP-EPC group). **c** Higher numbers of homed Ad5/β2AR EPCs than Ad5/EGFP EPCs were detected; *n* = 5 per group (*P < 0.01 versus Ad5/EGFP-EPC group). **d** Homing was detected in β2AR-overexpressing EPCs by using CM-DiI-labeled EPC tracing and FITC BS-1 lectin co-staining in frozen tissue sections. To demonstrate that the transfused EPCs that localized to the injured site were endothelial cells, null mice received FITC-labeled BS-1 lectin 30 min before tissues were harvested. *Ad5* adenovirus serotype 5, *DiI-ac-LDL* DiI-labeled acetylated low-density lipoprotein, *EGFP* enhanced green fluorescent protein, *EPC* endothelial progenitor cell, *FITC* fluorescein isothiocyanate, *PBS* phosphate-buffered saline, *β2AR* β2 adrenergic receptor

The carotid endothelium injury in mice was confirmed by Evans blue staining (Fig. 4a). To assess the capability of transplanted EPCs on endothelium recovery, we injected PB-derived EPCs into a nude mice model in which we caused wire-denuded carotid arteries. PBS, Ad5/β2AR-transduced EPCs, or Ad5/EGFP-transduced EPCs were injected into nude mice through the tail vein. Notably, compared with PBS, treatment with non-transduced EPCs or with Ad5/EGPF-transduced EPCs substantially increased re-endothelialization of denuded carotid arteries in nude mice; however, injecting the Ad5/β2AR-transduced EPCs resulted in a larger re-endothelialization area in the injured carotid arteries (82.1 ± 4.2 % versus 44.4 ± 6.5 % or 46.8 ± 4.3 % versus 26.6 ± 7.5 %, *n* = 5, *P* < 0.01; Fig. 4b). To determine whether labeled EPCs were able to home and incorporate into the sites of vascular endothelium injury, each mice was injected with 1 × 10^6^ CM-DiI-labeled EPCs after carotid artery injury. DiI-labeled EPCs were identified as red fluorescent cells. Data showed that there were more homing EPCs in the Ad5/β2AR-transduced EPC group than in the Ad5/EGFP-transduced EPC group that incorporated into the FITC-lectin-positive endothelial layer (Fig. 4c, d).

### The β2AR/Akt/eNOS pathway improves EPC function in vitro and the endothelial repair capacity of EPCs in vivo

The activations of Akt and eNOS are both important to determining the number and function of active EPCs. The activation of βARs in cardiac progenitor cells (CPCs) was previously shown to promote the proliferation and survival of CPCs and to be associated with the increased phosphorylation of Akt and eNOS. Therefore, we examined whether the β2AR/Akt/eNOS signaling pathway plays an important role in the proliferation, NO production, migration, and adhesion of EPCs. We used inhibitors, including ICI118,551 (a selective inhibitor of β2AR), LY294002 (an Akt inhibitor), and L-NAME (an eNOS inhibitor), to inhibit the activation of the β2AR/Akt/eNOS signaling pathway.

**Fig. 5:**
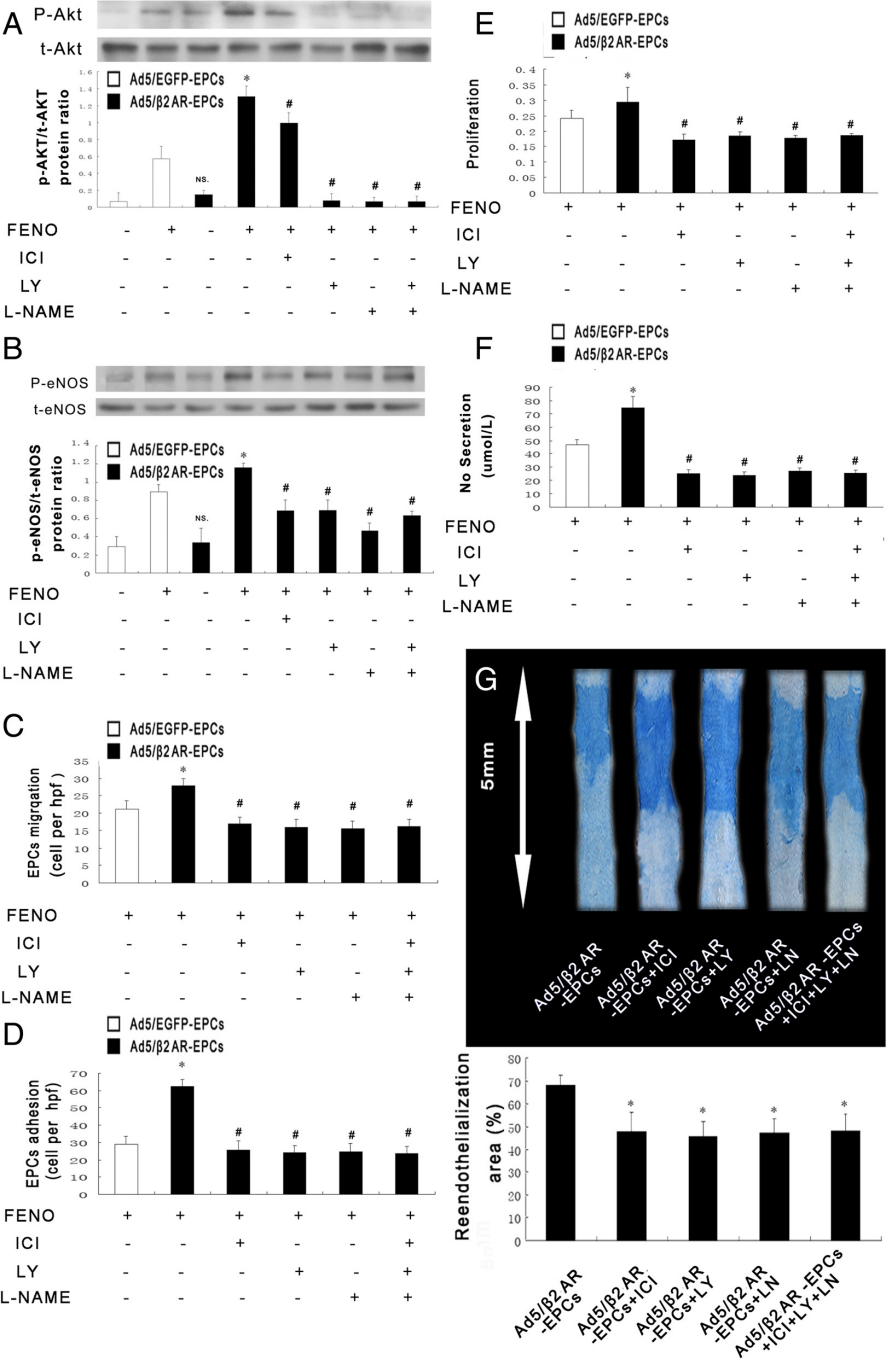
The β2AR/Akt/eNOS pathway improves EPC function in vitro and the endothelial repair capacity of EPCs in vivo. **a** Representative photograph and quantification analysis of Akt phosphorylation in EPCs (*P < 0.01 versus Ad5/EGFP-EPCs with FENO activation, ^#^ *P* < 0.05 versus Ad5/β2AR-EPCs with FENO activation, and NS versus Ad5/EGFP-EPCs; *n* = 5 per group). **b** Representative photograph and quantification analysis of eNOS phosphorylation in EPCs (*P < 0.01 versus Ad5/EGFP-EPCs with FENO activation, ^#^ *P* < 0.01 versus Ad5/β2AR-EPCs with FENO activation, and NS versus Ad5/EGFP-EPCs; *n* = 5 per group). **c** Quantification analysis of migration (*P < 0.01 versus Ad5/EGFP-EPCs with FENO activation and ^#^ *P* < 0.01 versus Ad5/β2AR-EPCs with FENO activation; *n* = 5 per group). **d** Quantification analysis of adhesion (*P < 0.05 versus Ad5/EGFP-EPCs with FENO activation and ^#^ *P* < 0.05 versus Ad5/β2AR-EPCs with FENO activation; *n* = 5 per group). **e** Quantification analysis of proliferation (*P < 0.05 versus Ad5/EGFP-EPCs with FENO activation and ^#^ *P* < 0.05 versus Ad5/β2AR-EPCs with FENO activation; *n* = 5 per group). **f** Quantification analysis of NO production (*P < 0.05 versus Ad5/EGFP-EPCs with FENO activation and ^#^ *P* < 0.05 versus Ad5/β2AR-EPCs with FENO activation; *n* = 5 per group). **g** The re-endothelialized area at day 3 after carotid injury in nude mice with transplantation of Ad5/β2AR-EPCs that were pre-treated with β2AR or Akt or eNOS inhibitors (*P < 0.01 versus Ad5/β2AR-EPCs; *n* = 5 per group). *Ad5* adenovirus serotype 5, *EGFP* enhanced green fluorescent protein, *eNOS* endothelial nitric oxide synthase, *EPC* endothelial progenitor cell, *FENO* fenoterol, *NO* nitric oxide, *NS* not significant, *β2AR* β2 adrenergic receptor

Following stimulation with FENO (10^−8^ M) for 6 h, the phosphorylation levels of Akt and eNOS were enhanced in the Ad5/β2AR-transduced EPCs compared with those in the Ad5/EGFP-transduced EPCs and in the non-transduced EPCs. Furthermore, the increases in Akt and eNOS phosphorylation that were observed in the Ad5/β2AR-transduced EPCs were inhibited by pre-incubation for 1 h with 0.3 μ M of ICI118,551, 10 μM LY294002 or 100 μM L-NAME (Fig. 5a, b). Interestingly, we also found that β2AR overexpression-mediated effects on proliferation, NO production, migration, and adhesion were all significantly inhibited by pre-treatment with ICI118,151, LY294002, and L-NAME (Fig. 5c-f). These results indicate that the β2AR/Akt/eNOS signaling pathway is at least partially responsible for the function of EPCs in vitro. We also investigated whether the β2AR/Akt/eNOS signaling pathway is associated with the re-endothelialization capacity of EPCs in vivo. In agreement with the in vitro results, pre-treatment with inhibitors of β2AR, Akt, and eNOS attenuated the enhanced re-endothelialization in the mice that were transplanted with Ad5/β2AR-transduced EPCs (Fig. 5g).

## Discussion

The present study demonstrates that β2AR expression on EPCs is involved in the restoration of damaged endothelium. Ad5/β2AR gene transfer treatment enhanced β2AR expression in EPCs and increased the capacity of EPCs to migrate, adhere, proliferate, and secrete NO in vitro and the re-endothelialization capacity of EPCs in vivo. Moreover, the increase in re-endothelialization capacity of EPCs is closely correlated with upregulation of the β2AR/Akt/eNOS pathway. Our present study is the first to demonstrate that β2ARs play a crucial role in regulating the vascular endothelial repair function of EPCs. At the molecular level, these effects were found to be at least partially associated with β2AR/Akt/eNOS pathway activation.

Endothelial dysfunction is known to play pivotal roles in degenerative vascular disease. Restoration of endothelial integrity is an important technique that can be used to cure vascular disease. Since Asahara et al. [19] first cultivated EPCs, numerous experiments have shown that EPCs mediated endothelial restoration in many processes, suggesting that they may also be useful for treating vascular injury. The term EPCs has been applied interchangeably to a variety of cell populations by different investigators, suggesting that EPCs are not a single type of cell population. The definition of EPCs remains controversial. Many studies have attempted to identify cell surface markers that are unique to EPCs and to distinguish them from mature ECs and from myeloid-monocytic cells; however, these attempts have met with little success. Broadly speaking, two approaches to identify EPCs have predominated: (1) identification of cells bearing surface markers that indicate both cellular naïveté and endothelial origin and (2) inference of the presence of endothelial precursors with a given cell population by the identification of cells bearing mature endothelial characteristics after a period of culture under angiogenic conditions [20]. In our experiment, according to the method of Hur et al. [14], isolated MNCs were resuspended by using the EGM-2 BulletKit system. After 5 to 7 days, attached cells were elongated and had a spindle shape. The recognition of cell surface antigen markers by flow cytometric analysis has confirmed the cells as early EPCs, consistent with the results of other studies [12, 21].

Maintenance of the normal number and function of EPCs in the systemic circulation is now known to be an important novel endogenous vascular repair factor. In animal and clinical research, transplanted EPCs can home to impaired arteries, promoting re-endothelialization and reducing neointima formation. Various molecules may be involved in the process of EPC homing. EPCs and ECs have previously been shown to express both β1ARs and β2ARs [8, 9]. β2ARs are G protein-coupled receptors that can induce cell proliferation and promote cell survival in many tissues, and stimulation of endothelial β2ARs has been shown to activate eNOS and release of NO in human umbilical vein endothelium [22]. Previous studies have shown that EPCs and ECs harvested from β2AR-knockout (β2AR-KO) mice are impaired in their abilities to migrate and stimulate network formation on Matrigel in vitro [8, 9]. Moreover, when β2AR-KO ECs and EPCs were injected into ischemic hind limbs, no significant amelioration of neovascularization was noted [8, 9]. However, whether β2ARs mediate the capacity of EPCs to re-endothelialize damaged arteries following an injury has never previously been tested. Given the results described in prior reports, we hypothesized that β2AR overexpression in human EPCs would result in the functional enhancement of EPCs in vitro and that β2AR gene upregulation would contribute to the restoration of endothelial injury in vivo.

To address these assumptions, we first investigated the effect of β2AR overexpression on the proliferation, migration, adhesion, and NO secretion of EPCs. We found that when EPCs were stimulated with FENO, the rates of proliferation and migration were significantly higher in EPCs that overexpressed β2ARs, indicating that FENO-induced functions were enhanced by β2AR overexpression in EPCs. Furthermore, our results showed that TNF-α-induced adhesion and NO secretion were enhanced by β2AR overexpression in EPCs. Given the close association between β2AR and EPC function, deeper insight into the contribution of β2AR expression in EPCs to accelerated re-endothelialization may be of clinical importance for the treatment of CVDs. We investigated the effect of infusing cultured PB-derived EPCs in a previously described nude mouse model of carotid artery injury. As shown in Fig. 4b, β2AR overexpression in the infused EPCs resulted in a significantly larger area of re-endothelialization, suggesting that β2AR signaling might be an important molecular mechanism that contributes to enhanced re-endothelialization by EPCs. Notably, the SDF-1α/CXCR4 interaction plays an important role in the regulation of a variety of cellular functions, including cell migration, proliferation, survival, and angiogenesis. Previous studies have demonstrated that the SDF-1a/CXCR4 axis is crucial for the therapeutic integrity of EPCs and their ability to home to injured vessels. For example, investigators showed that SDF-1a was involved in ischemia-mediated mobilization and homing in EPCs after vascular injury [18]. It has also been reported that CXCR4 expression restored the functions of EPCs in hypertensive patients [23]. Our data showed that stimulation with β2AR promoted EPC function and significantly increased SDF-1a and CXCR4 expression in cultured EPCs, especially in EPCs that overexpressed β2AR. These data collectively suggest that β2AR is an important potential therapeutic target and that β2AR-mediated EPC functions are dependent, at least in part, on the production of SDF-1a/CXCR4 and may otherwise be equal to the SDF/CXCR4 axis in the biology of EPCs. Therefore, our results indicate that transplantation of EPCs that have enhanced β2AR expression could be used as a novel therapy for treating vascular endothelial injuries.

In regard to how β2AR gene transfer modulates re-endothelialization, several lines of evidence have shown that activation of β2ARs promoted the proliferation and survival of cardiac progenitor cells in association with increased eNOS phosphorylation [24, 25]. Akt, a multifunctional regulator of cell survival, is a downstream effector of phosphatidylinositol-3 kinase (PI3K). It is widely accepted that activating Akt phosphorylation stimulates eNOS phosphorylation at Ser1177 and increases endothelial NO production, which is involved in the mobilization of stem and progenitor cells [26]. Our current data demonstrate that this signal paradigm also exists in EPCs because β2AR stimulation increased the phosphorylation of Akt and eNOS, which is consistent with increased EPC proliferation, migration, and adhesion function in vitro and with accelerated re-endothelialization in vivo. These cellular effects are blocked by β2AR/Akt/eNOS inhibition. Furthermore, we upregulated the expression of β2AR via Ad5/β2AR gene transfer and found that the increased re-endothelialization capacity that was induced in the cultured EPCs that overexpressed β2AR was blocked by the β2AR inhibitor ICI111, 181; the Akt inhibitor LY294002; and the eNOS inhibitor L-NAME. These data strongly support the hypothesis that β2AR plays a crucial role in the regulation of vascular repairs by circulating EPCs. We believe that this study is also the first to provide data indicating that mechanisms involving β2AR signaling underlie the functions of EPCs in vitro and their capacity to re-endothelialize injuries in vivo.

The present study has some limitations. First, although we used a β2AR inhibitor to explore the important functions of EPCs in the vitro and in vivo experiments, we did not focus on exploring the functional impairments that were observed in the β2AR-KO EPCs during homing to impaired arteries. Second, β2AR gene transfer results in accelerated re-endothelialization after vascular injury, but the adenovirus-mediated β2AR gene overexpression should be verified to determine its clinical safety. Finally, we used a relatively simple model in nude mice to demonstrate that β2AR gene transfer resulted in EPCs with enhanced repair capacities. Whether the β2AR/Akt/eNOS signaling pathway contributes more or less to EPCs to mediate endothelial repair relative to the contributions of other signaling pathways needs to be further investigated.

## Conclusions

Our study is the first to provide direct evidence that β2AR expression in EPCs is an important molecular target for therapeutic studies and that β2AR activation may be responsible for accelerated EPCs homing during injury. More importantly, our study demonstrates that overexpressing the β2AR gene in EPCs results in rapid re-endothelialization and largely improved post-injury vascular repairs and that these processes are mediated by the β2AR/Akt/eNOS signaling pathway. Stem cell therapies have shown great potential as strategies aimed at increasing the efficiency of vascular regeneration; therefore, β2AR gene may become a novel therapeutic molecular target in clinical studies aimed at improving cardiovascular care. These scientific questions deserve further investigation.

## Abbreviations

Ad5: adenovirus serotype 5
BSA: bovine serum albumin
CVD: cardiovascular disease
DAPI: 4‘,6-diamidino-2-phenylindole
DiI-ac-LDL: DiI-labeled acetylated low-density lipoprotein
EBM-2: endothelial basal medium-2
EC: endothelial cell
EGFP: enhanced green fluorescent protein
eNOS: endothelial nitric oxide synthase
EPC: endothelial progenitor cell
FBS: fetal bovine serum
FENO: fenoterol
FITC: fluorescein isothiocyanate
GAPDH: glyceraldehyde 3-phosphate dehydrogenase
HUVEC: human umbilical vein endothelial cell
ICI: ICI118,551 (a selective inhibitor of β2AR)
KDR: kinase-insert domain receptor
KO: knockout
LY: LY294002 (an Akt inhibitor)
mAb: monoclonal antibody
MNC: mononuclear cell
MOI: multiple of infection
NO: nitric oxide
PB: peripheral blood
PBS: phosphate-buffered saline
PCR: polymerase chain reaction
PE: phycoerythrin
PFA: paraformaldehyde
SDF-1a: stromal cell-derived factor 1a
TNF-α: tumor necrosis factor-α
VEGF: vascular endothelial growth factor
vWF: von Willebrand factor
β2AR: β2 adrenergic receptor
